# A Predictive Model for Patient Census and Ventilator Requirements at Individual Hospitals During the Coronavirus Disease 2019 (COVID-19) Pandemic: A Preliminary Technical Report

**DOI:** 10.7759/cureus.8501

**Published:** 2020-06-08

**Authors:** Richard H Epstein, Franklin Dexter

**Affiliations:** 1 Anesthesiology, University of Miami Miller School of Medicine, Miami, USA; 2 Anesthesiology, University of Iowa, Iowa City, USA

**Keywords:** covid-19, forecasting, length of stay, ventilators

## Abstract

During the initial wave of the coronavirus disease 2019 (COVID-19) pandemic, many hospitals struggled to forecast bed capacity and the number of mechanical ventilators they needed to have available. Numerous epidemiological models forecast regional or national peak bed and ventilator needs, but these are not suitable for predictions at the hospital level. We developed an analytical model to assist hospitals in determining their census and ventilator requirements for COVID-19 patients during future periods of the pandemic, by using their data. This model is based on (1) projection of future daily admissions using counts from the previous seven days, (2) lengths of stay and duration of mechanical ventilation, and (3) the percentage of inpatients requiring mechanical ventilation. The implementation is done within an Excel (Microsoft, Redmond, WA) workbook without the use of add-ins or macro programming. The model inputs for each currently hospitalized patient with COVID-19 are the duration of hospitalization, whether the patient is currently receiving or has previously received mechanical ventilation, and the duration of the current ventilation episode, if applicable. Data validity and internal consistency are checked within the workbook, and errors are identified. Durations of care (length of hospital stay and duration of mechanical ventilation) are generated by fitting a two-parameter Weibull distribution to the hospital’s historical data from the initial phase of the pandemic (incorporating censoring due to ongoing care), for which we provide source code in the R programming language (R Foundation for Statistical Computing, Vienna, Austria). Conditional distributions are then calculated using the hospital’s current data. The output of the model is nearly instantaneous, producing an estimate of the census and the number of ventilators required in one, three, and seven days following the date on which the simulation is run.

Given that the pandemic is ongoing, and a second surge of cases is expected with the reopening of the economy, having such a tool to predict resource needs for hospital planning purposes has been useful. A major benefit to individual hospitals from such modeling has been to provide reassurance to state and local governments that the hospitals have sufficient resources available to meet anticipated needs for new COVID-19 patients without having to set aside substantially greater numbers of beds or ventilators for such care. Such ongoing activity is important for the economic recovery of hospitals that have been hard-hit economically by the shutdown in elective surgery and other patient care activities. The modeling software is freely available at https://FDshort.com/COVID19, and its parameters can easily be modified by end-users.

## Introduction

Multiple groups have created epidemiological models for forecasting, at the regional or national level, the numbers and peak occurrence of cases of coronavirus disease 2019 (COVID-19), admissions, deaths, and mechanical ventilator requirements [1]. These models are useful for policy decision-making but are inadequate for individual hospitals because the prediction limits are large, and the simulations generally do not take into account community factors (e.g., age distribution, local socioeconomic factors) that may influence the distribution of patients among hospitals [2-5]. Further, these epidemiological models do not address specific patient management requirements of individual hospitals on a short-term basis. For example, during the COVID-19 pandemic, each hospital has needed to estimate the number of hospital beds and ventilators they needed. A few days of advance warning is required to make planned conversions of hospital wards to allow for isolation facilities for COVID-19, to repurpose anesthesia machines to be used as intensive care ventilators, to modify call schedules, and to potentially curtail elective surgical cases to increase hospital capacity.

Some regional healthcare models of the pandemic have also been created, such as the Monte-Carlo simulation approach developed at the University of Pennsylvania [6]. These models are useful for facility management planning in advance of the expected surge [e.g., stopping elective surgery, increasing ward bed and intensive care unit (ICU) capacity, accelerating completion of a new hospital wing], but not for daily operational needs. To apply the University of Pennsylvania model to an individual hospital, estimates must be provided for unknown parameters, such as, for example, the percentage of a region’s patients who will go to that hospital and the proportion of the regional population that will remain sufficiently isolated so that they avoid infection.

In this technical report, we describe a different approach: an analytical model for individual hospitals using their data to help plan for their requirements related to patient census and mechanical ventilators. Our model combines (1) estimation of the daily numbers of admissions among patients with COVID-19 at the local hospital, (2) local data for lengths of stay and duration of mechanical ventilation among patients previously admitted to the hospital, and (3) the percentage of inpatients who require mechanical ventilation. Our software application is available for download to readers using Excel (Microsoft, Redmond, WA), at https://FDshort.com/COVID19. The model is implemented as a simple workbook with no requirements for either add-ins or macros requiring Visual Basic for Applications (Microsoft) modules. The underlying mathematics will apply both to hospitals experiencing surges in their COVID-19 admissions (i.e., during acceleration phases) and to hospitals managing a few admissions daily (e.g., when the hospital and ICU census of COVID-19 patients is falling). Having a method to predict the extent to which future resource needs are dropping was useful at the University of Iowa for activities such as returning anesthesia machines set aside to ventilate ICU patients to the operating room and to ensure that an appropriate amount of resources are available for future patients with COVID-19 while allowing elective surgery and procedures to continue. The latter is especially important for hospitals trying to recover from economic setbacks resulting from the curtailing of elective patient care during the first wave of the pandemic. State and local requirements to set aside an excessive number of ICU and ward beds for potential patients will interfere with such efforts. It has been observed that bed and ventilator needs are dominated, in the short-term, not by the arrival of new patients with the disease, but by the long hospital length of stays and ventilator days among inpatients, especially following the peak of COVID-19 admissions. For many hospitals with few (<10) daily admissions of COVID-19 patients and even fewer ICU admissions requiring mechanical ventilation, the workbook is designed to assist in just-in-time resource management. Under both scenarios, the model uses the hospital’s own data to help predict resource needs and guide ongoing activities.

Because the COVID-19 pandemic is still evolving worldwide, there are a limited number of complete (uncensored) datasets describing the entire time course of severe illness (e.g., length of hospital stay, length of ICU stay, probability of mechanical ventilation, duration of mechanical ventilation). Published data related to the duration of treatments have typically only reported on cases where the outcomes of interest are evaluable. In contrast, our modeling incorporates censoring in the estimation of the distributions describing lengths of stay and duration of mechanical ventilation. As we will demonstrate, there are considerable differences among hospitals, and making use of each hospital’s data is necessary. This is not a weakness of our approach, rather a strength, in that it reveals this reality and reinforces the idea that using statewide planning models to guide decisions at individual hospitals will result in poor hospital resource planning. Because the data requirements to run the model are small and can be produced easily by many hospitals’ information technology analysts, we think our model will have utility in the ongoing management of the pandemic.

## Technical report

The University of Miami Institutional Review Board approved the study (ID NO: 0200682) on May 29, 2020, with a waiver of consent and a full HIPAA waiver of authorization. The Institutional Review Board of the University of Iowa determined that the study was non-human subjects research on May 19, 2020, because only aggregate data were used from its patients. Electronic health record data used were from the main hospital of the University of Miami UHealth Tower (UHT), between March 1, 2020, and May 16, 2020, and from the University of Iowa Hospitals and Clinics (UIHC) between March 1, 2020, and May 8, 2020. Patients were classified as having COVID-19 if they had a positive reverse transcriptase-polymerase chain reaction test for severe acute respiratory syndrome coronavirus 2 (SARS-CoV-2).

Data requirements

User input to the model requires three data elements for each current inpatient with COVID-19 on the date the simulation is run: (a) days in the hospital since the admission date, (b) days of mechanical ventilation if ongoing, otherwise zero days, and (c) whether or not the patient currently is or previously was on mechanical ventilation. Also, the fraction of patients with COVID-19 who required mechanical ventilation historically needs to be supplied. The data would be extracted from the hospital’s electronic health record and then processed to the format required for pasting into the model workbook. For example, in our uploaded Excel workbook, we have provided descriptions of the fields necessary to build such a report in Epic (Epic Systems, Verona, WI). In the modeling workbook, error-checking is applied to each of the entered data elements. For example, negative numbers, fractions where integers are required, or numbers incorrectly formatted as a string (e.g., the text characters “12” instead of the number 12) are detected by Excel formulas and the individual datum with errors is highlighted. Recommendations are provided automatically using Excel conditional statements.

To facilitate the use of the uploaded Excel workbook and to avoid inadvertent deletion or alteration of required cells, the workbook and worksheets are protected. However, there are no passwords on purpose. To see the formulas or edit the workbook, readers should select the “Review” item in the Excel ribbon and then click on the “Unprotect Worksheet” icon.

Lists of lengths of stays for patients who required or who did not require mechanical ventilation and the duration of mechanical ventilation are necessary to generate the parameters of the distribution curves describing these parameters (see next section). If the patient’s care is ongoing (e.g., still in the hospital or on mechanical ventilation), those data also are needed. Code in the programming language R (R Foundation for Statistical Computing, Vienna, Austria) is supplied, which will generate the distribution function parameters.

Parameter values

Guan et al. have reported the proportions of patients hospitalized with severe or non-severe disease, listed in age categories [7]. However, at neither hospital we studied did we find a relationship among hospitalized patients between patient age and the likelihood of mechanical ventilation (e.g., at UHT, 29.5% of those <65 years, 31.7% of those of ages 65-74, and 30.2% of those 75 years or older). Thus, for purposes of our modeling length of stay, we characterized patients as having a severe disease if they required mechanical ventilation, and non-severe otherwise. In our modeling at UHT, we used the observed prevalence of mechanical ventilation among admitted patients of 30.3%. This value will vary among hospitals, influenced in part by the fraction of patients with mild to moderate disease who are managed as outpatients [8].

We did not include gender in the model because this did not influence outcomes among admitted patients. For example, Guan et al. reported that women represented 41.8% of patients with a non-severe disease and 42.2% of patients with severe disease [7]. At UHT, there was also no influence of gender on the length of stay (95% CI of difference: -2.9 to 3.8 days, p=0.77 by Student’s t-test) or on the probability of requiring mechanical ventilation [25% (16/64) females vs. 34% (31/90) males, p=0.22 by Fisher’s exact test]. 

Modeling durations using Weibull distributions

We used three probability distributions for durations in our modeling: (1) length of stay in the hospital among COVID-19 patients with severe disease (i.e., not requiring mechanical ventilation), (2) length of stay in the hospital among such patients with non-severe disease, and (3) the duration of mechanical ventilation when instituted. During the initial model development, we used published data for the medians and interquartile ranges (Table 1) [7,9]. 

**Table 1:** Previously published parameter values (shape, scale) for the Weibull distribution related to durations of treatment MAE: mean absolute error

Parameter	Disease	Shape	Scale	MAE (days)	Median (days)	25^th^ Percentile (days)	75^th^ Percentile (days)
Hospital length of stay [7]	Non-severe	6.183	12.23	0.2	11.0	10.0	13.0
Hospital length of stay [7]	Severe	4.023	15.67	0.4	13.0	11.5	17.0
Ventilator days [9]	Severe	2.269	6.93	0.3	5.0	4.0	8.0

Each of these distributions was fit to a Weibull distribution. We used this distribution for several reasons. First, given that we only had the 25th, 50th, and 75th percentiles for the durations available from the literature, the choice of the distribution used was not of major importance. The Weibull formula is flexible, as it can be fit to exponential or skewed distributions, depending on the value of the shape parameter [10]. Second, the Weibull distribution is used routinely for estimation of the time to removal of the tracheal tube in patients in intensive care and when undergoing general anesthesia, providing us with estimates of appropriate starting conditions for the shape parameter [11,12]. Third, the Weibull distribution has an analytical conditional distribution function [13]. The importance of this feature is that it allowed for computation of the probability of a patient remaining in the hospital or on a ventilator, conditional on the number of days the patient has already been in the hospital or on the ventilator. Because the conditional distribution is analytical, these calculations do not require the use of macro functions comprised of software written using Visual Basic for Applications. Many hospitals block the downloading of files containing software because these can contain malicious code; moreover, such reliance on a macro-enabled workbook would limit the distribution of the simulation package. Thus, we avoided the use of executable code in our model. Finally, subsequent to the initial submission of our manuscript, a paper was published by Lewnard et al. in which the Weibull distribution was used to model the duration of hospitalization of COVID-19 patients, similar to what we describe below for right-censored data from UHT [14].

**Figure 1:**
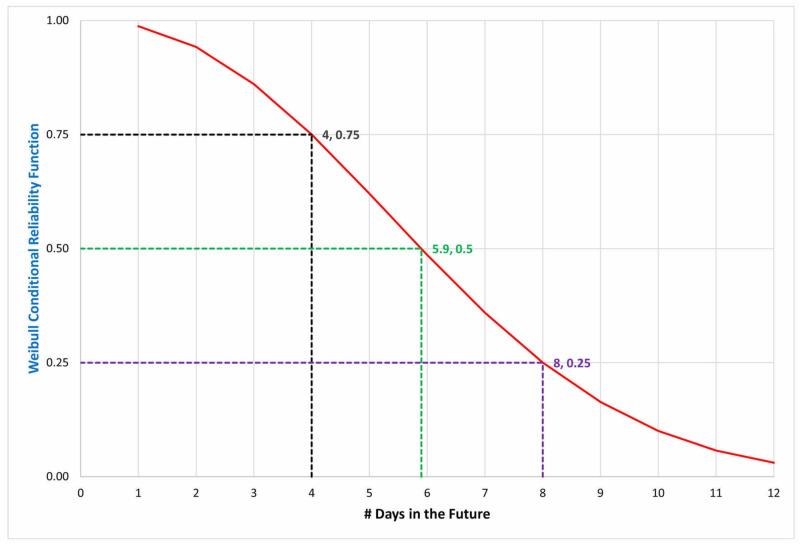
Determination of the shape and scale parameter for the duration of mechanical ventilation Determination of the shape and scale parameter for the duration of mechanical ventilation using previously published data [9]. The reported statistics were a median (50th percentile) = five days, 25th percentile = four days, and 75th percentile = eight days. A Weibull distribution curve (red line) was fit to minimize the sum of the absolute errors between the predicted and reported values at those 3 percentiles (dashed lines). The shape was 2.269, and the scale was 6.93. The fit curve matches the published data, with a mean absolute error of only 0.3 days, where 0.3 = ((4-4) + (5.9-5) + (8-8))/3). This figure can be compared with the conditional distribution in Figure 2

Figure 1 presents an example describing the use of the Weibull conditional probability function when only the median and interquartile values are available. The figure shows the probability distribution of days remaining on the ventilator for a patient who has been receiving mechanical ventilation. To create the shape and scale parameters for this distribution function, we used Excel Solver using the generalized reduced gradient (GRG) Nonlinear optimization option. We minimized the mean absolute error for the 25th, 50th, and 75th percentiles, based on the published values for the distribution of ventilator duration [9]. From multiple initial starting conditions for the shape and scale parameters, the parameter estimates minimizing the error were shape = 6.930 (unitless) and scale = 2.269 (units of days). The error term for the best fit was 0.3 days. The fit (reported) values for the 25th, 50th, and 75th percentiles were 4.00 (4) days, 5.90 (5) days, and 8.00 (8) days, respectively.

**Figure 2:**
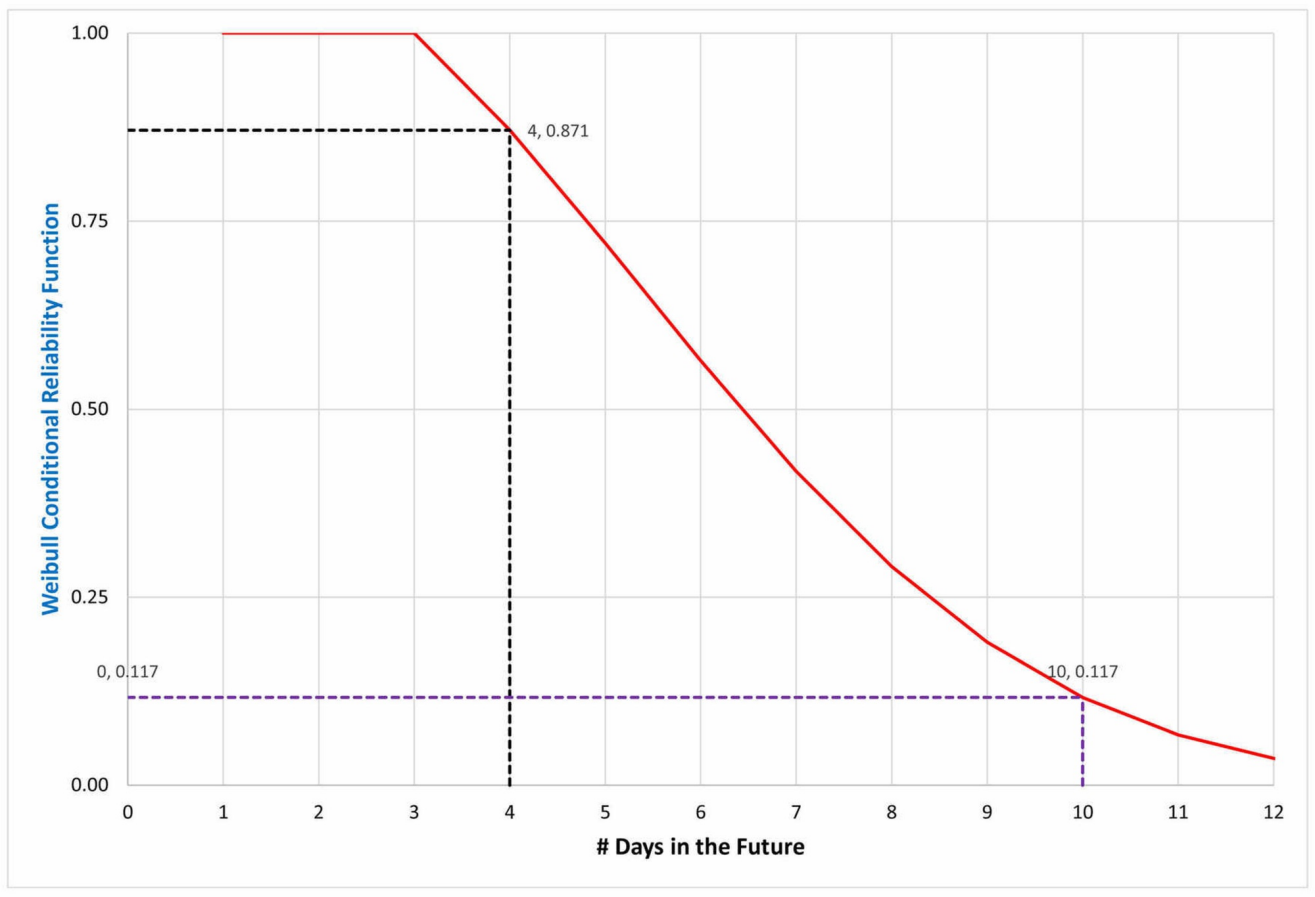
Probability curve of remaining on the ventilator for an additional one and seven days after already having been ventilated for three days The Weibull shape and scale parameters for the fit curve (red line) are as described in Figure 1. There is an 87.1% chance that the patient would still be on the ventilator after one additional day (four days after the start of ventilation, black dashed line), but only an 11.7% chance after seven days (10 days after the start of ventilation, purple dashed line). These durations of treatment primarily do not reflect weaning from mechanical ventilation and extubation, but rather death, given the high mortality of patients with COVID-19 whose respiratory failure progresses to the point of requiring tracheal intubation. This figure can be compared with the fitted distribution in Figure 1

Figure 2 shows the conditional probability of remaining on the ventilator seven days after already being ventilated for three days. There is an 87.1% chance that the patient will still be ventilated the next day, but only an 11.7% chance of this seven days later, i.e., 10 days after the start of mechanical ventilation.

**Figure 3:**
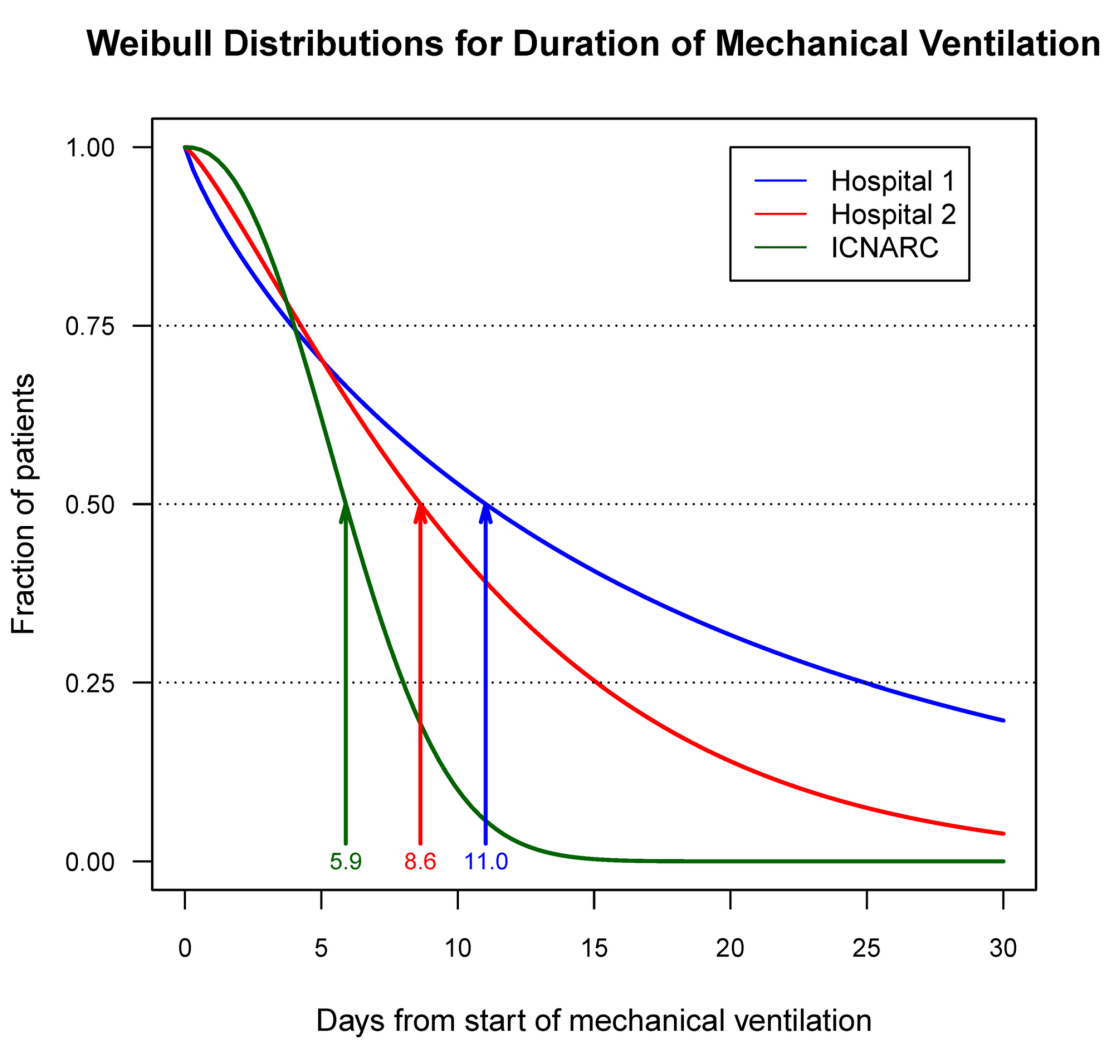
Weibull distribution curves for the duration of mechanical ventilation using data from the two US hospitals studied* Since it was not the objective of this study to compare the two hospitals, their identities have been hidden on this graph. On the x-axis, the number of days from the onset of mechanical ventilation (day zero) is displayed. On the y-axis, the fraction of patients remaining on mechanical ventilation at each time is shown. The fraction decreases over time according to the number of patients who are extubated and who expire. While 75% of the patients at hospital 1 (green line), 2 (red line), and from the published ICNARC report (blue line) remained intubated for similar durations (4.0, 4.2, and 3.9 days, respectively), there was an increasing spread between groups in the duration of time at which 50% and 25% of the patients remained extubated [9]. The arrows on the graph represent the median duration of mechanical ventilation in the three groups, with corresponding colors. For the 25% quartile, these times were 8.0, 15.1, and 15.0 days, respectively. This figure highlights the need for hospitals to use their own data in generating distribution curves describing the clinical course of the patients ^*^From published median and interquartile range data from reference 9 [9] ICNARC: Intensive Care National Audit and Research Centre

During initial development, since we did not have sufficient numbers of patients with COVID-19, we used the Weibull distributions fit to the published median and interquartile range values for the various durations [7,9]. However, progressively during the acute phase of the pandemic, it became apparent that data at UHT and UIHC did not match the published data from China and Europe regarding the duration of hospitalization and mechanical ventilation [8,9]. For example, predicted durations of mechanical ventilation differed substantively among the two hospitals and the published reports (Figure 3). These differences imply that hospitals need to use their local data. Therefore, our evaluation of the predictive accuracy of modeling at UHT (below) was based on the Weibull fits of lengths of hospitalization for patients with severe and non-severe diseases and durations of mechanical ventilation from that hospital.

Considerations related to the number of hospitalized inpatients at the time of modeling

For each day that a hospital uses the model (i.e., the Excel workbook), there will be a census of patients in the hospital, each present for a varying number of days. There also will be, for each hospitalized patient, the preceding number of days of mechanical ventilation, equaling zero for most patients. In determining the hospital census between one day and seven days later (i.e., the prediction horizon), the expected outcomes of these patients need to be determined. The probability for hospital length of stay was based on the conditional probability from the Weibull distribution based on the number of previous days of hospitalization. The distribution used was according to whether the patient had undergone mechanical ventilation at the start of the date on which the new model was run. Note that for a patient who underwent mechanical ventilation, the length of stay would have been from non-severe patient distribution until the date that mechanical ventilation started. Subsequently, severe patient distribution would be used. Similarly, the conditional probability for the duration of mechanical ventilation among ventilated patients was based on the number of previous continuous days of mechanical ventilation.

Considerations related to the number of newly hospitalized inpatients over the next week

Over the interval defined by the prediction horizon, additional patients will be admitted to the hospital. To account for these new patients, we explored four different estimation methods, based on the actual admissions over the previous seven days. These included linear and log-linear regression, the median number of admissions, and the number of admissions from the day immediately preceding the date of the modeling.

If one uses the census information to infer the number of admissions from previous days (e.g., a patient with a duration of hospitalization of one day was admitted the day before, a patient with a duration of hospitalization of seven days was admitted seven days ago), there will be an error introduced from patients who died or who were discharged during the previous seven days. For example, if a patient admitted five days ago expires on the day before the current model is run, there will be one fewer calculated admission from five days ago. However, that number is small, given the overall high survival rate among all admitted patients and the prolonged distribution of their lengths of stay. For example, using parameter values estimated from Guan et al., among the patients with non-severe disease, approximately 0% and 3% would have been discharged within one day and one week, respectively. Among the patients with severe disease, the incidences are 0% and 4%, respectively. The probability of discharge for patients with severe disease is higher not because they are recovering faster, but rather because they have a higher risk of death. For those using our workbook, we provide a mechanism to allow overriding the automatically calculated number of admissions with the actual numbers.

Calculation of admissions over the seven days of the model forecasts

For log-linear regression, using the number of patients hospitalized on each of the days prior to the model being run, the slope and intercept of the linear regression line are estimated using days for the x-axis and the log number of admissions for the y-axis. During the early phase of census growth, there might be days with no admissions; this would create an error because the log(0) is not defined. Thus, we force the minimum number of admissions on each day as one patient. During the acute phase of the COVID-19 crisis, it would be unlikely to have days with no admissions. From the estimated intercept and slope in the log scale, we forecast the number of patients admitted on each of the zero, one, … seven days in the future. For linear regression, we follow the same steps as above except that an arithmetic scale is used for the y-axis, and projected admissions less than one (e.g., as could occur during the waning phase of an acute COVID-19 patient surge) are converted to one. For the median method, the median of the previous seven days' admissions is used; this will always be an integer because the number of data points is odd. Finally, for the previous day estimate, we simply used the number of admissions that occurred on the day before the model is run.

We calculated the predicted number of admissions during the dates one, three, and seven days in advance using the four different methods and determined the error on each future date compared to the actual number of admissions on each simulation date between March 10, 2020, and May 9, 2020 (Table 2). Because these predictions are made at the start of the modeling day (i.e., day zero), include an estimate for that day, and are for all the admissions on the last date of the prediction horizon (i.e., by 11:59 PM), the effective numbers of days in the prediction horizon are two, four, and eight days, respectively. We calculated the absolute value of the difference between the actual number of admissions on the future date of the predictions and that predicted by the model. Our preference was to forecast using the median of previous days’ admissions because it is (1) simple to compute, (2) does not require adjustments for negative or zero predicted admissions, and (3) would be less influenced by outliers than other methods (e.g., if there were an unusually large number of admissions due to transfers from a nursing home). To determine if the median was a reasonable choice, we compared the absolute errors in days from the other methods to the absolute errors from the median method and computed the mean and standard error. We then computed two-sided t-tests to determine if the difference between the pairs of comparator methods was statistically different from zero days. Absolute errors compared to actual admissions for the log-linear and linear methods were larger than the absolute error using the median method for prediction horizons of one, three, and seven days. The error from the previous day method was larger than the method for the three-day prediction horizon but equivalent for the one- and seven-day prediction horizons. Overall, the median method had the best performance and was thus selected for the resource prediction model.

**Table 2:** Comparison of absolute errors relative to actual admissions from various methods of predicting admissions vs. using the median of the previous seven days at the University of Miami UHealth Tower *P-values were calculated using the two-sided Student t-test comparing the mean difference to zero

Difference between the absolute error in admissions from the prediction method and the median method				
Prediction horizon (days)	Admission prediction method	Mean	Standard error	P-values*
1	Log-linear regression	0.7	0.21	0.002
	Linear regression	0.67	0.18	<0.001
	Previous day	0.3	0.15	0.052
3	Log-linear regression	0.4	0.17	0.017
	Linear regression	0.49	0.15	0.002
	Previous day	0.22	0.15	0.14
7	Log-linear regression	0.87	0.28	0.004
	Linear regression	0.66	0.18	<0.001
	Previous day	0.13	0.16	0.402

Calculating the number of newly admitted patients who will still be present on day seven of the model forecast

For each day in the future, we calculate the expected number of newly admitted patients who will be present up to seven days from the start of the model. For example, for patients admitted on day three, we need to determine how many of them will still be present four days later (i.e., day seven). The number of such patients is calculated as follows: (probability of severe disease) × (probability of length of stay of four days or more from the Weibull distribution among those patients) + (1 - probability of severe disease) × (probability of length of stay of four days or more from the corresponding Weibull distribution among those patients with non-severe disease). We infer the presence of severe disease based on the prior or current use of mechanical ventilation at the time of the simulation (i.e., probability of severe disease = 1 or 0). The severity of the disease is determined at the time the model is run because the occurrence of future ventilation would not be known at the time of the forecast.

Calculating the total number of forecasted beds and ventilators one day, three days, and seven days in the future

The total forecasted beds and ventilators used at prediction horizons of one day, three days, or seven days are calculated by summing up the forecasts for patients currently in the hospital plus those expected to be hospitalized over the coming three or seven days, including day zero, depending on the selected prediction horizon (i.e., the future period = “three days or Next week” in the Excel workbook). To reiterate, the reader can download the described workbook from https://FDshort.com/COVID19.

Estimating the predictive error of the model

**Figure 4:**
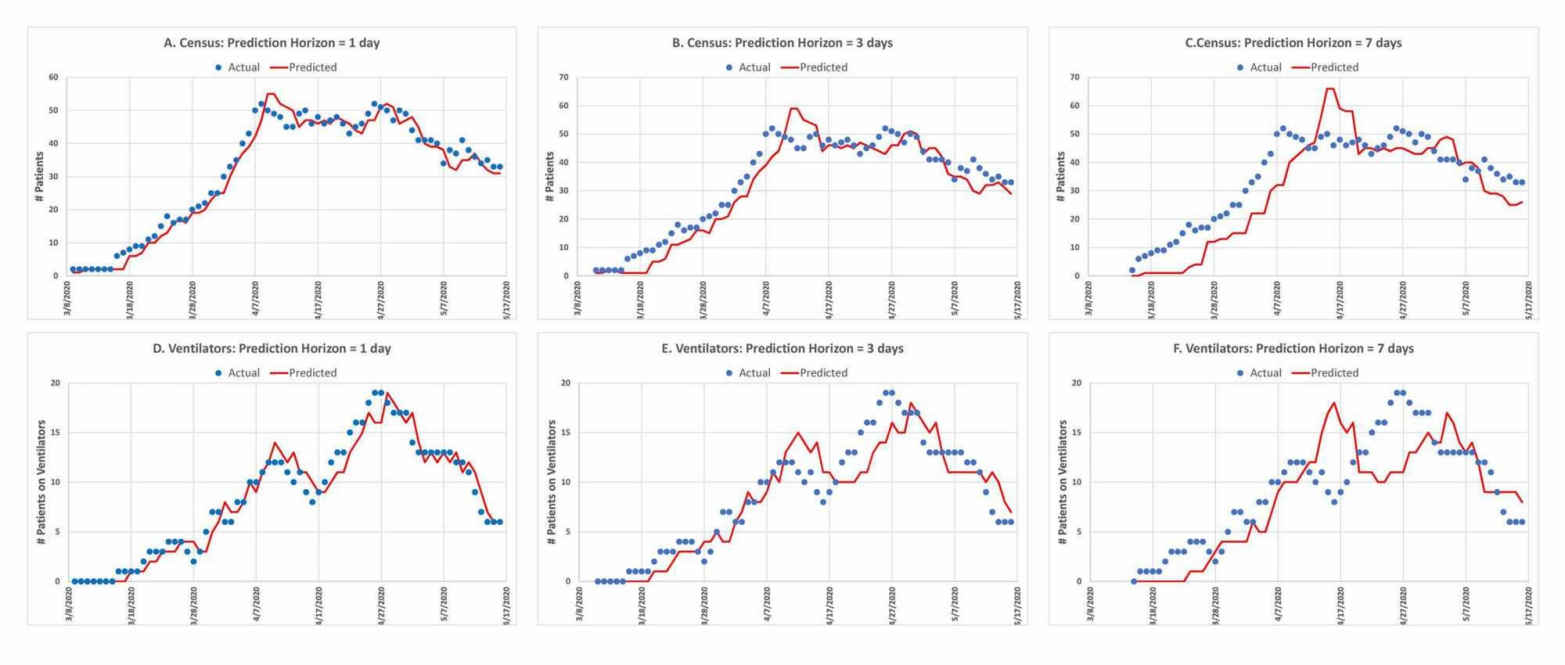
Predictions of the patient census and the number of patients receiving mechanical ventilation The blue dots represent the actual values and the red line the modeled predictions for patients at the University of Miami UHealth Tower. The top three panels, A-C, are for prediction horizons of one, three, and seven days for the hospital census, respectively. The bottom three panels, D-F, are similarly for the number of patients requiring mechanical ventilation

We evaluated the predictive error of the model using data from UHT for the COVID-19 pandemic through May 16, 2020. For each patient admitted to the hospital with documented COVID-19, we extracted the admission date and, when applicable, the discharge date, the date of start and end of mechanical ventilation, and the date of death. For each date between March 1, 2020, and May 16, 2020, we calculated the number of patients with COVID-19 who were an inpatient for at least part of the day (the hospital census), the number of patients undergoing mechanical ventilation for at least part of the day, and the number of new admissions. The actual number of admissions for each day was used for the predictions, as this value was easily obtainable and more accurate than imputing the number of admissions from the number of hospitalized days. The model reasonably matched the actual data for patient census and the number of patients on mechanical ventilation each day for the one-, three-, and seven-day future predictions (Figure 4). During the initial phase of COVID-19 admissions, the predictions tended to underestimate the resource requirements. That was expected, given that the number of admissions each day was increasing, and the estimate of new admissions was based on the previous seven days. After reaching the plateau of admissions (approximately April 8), there was an initial overshoot in resource predictions. That also was expected, due to the decrease in the admission rate and the use of the data from the previous seven days.

Model performance was reasonable, with small overall mean absolute error per day in predictions for both census (<1.25 patients per day) and ventilators (<0.5 ventilators per day) (Table 3).

**Table 3:** Mean absolute errors per day for hospital census and ventilators in use for prediction horizons of one to seven days among patients with COVID-19 at the University of Miami UHealth Tower SE: standard error

Absolute error per day for beds and ventilators				
Resource	Prediction horizon (days)	No. of predictions	Mean	SE
Census	1	69	1.24	0.12
	3	67	1.01	0.1
	7	63	0.91	0.08
Ventilators	1	69	0.49	0.05
	3	67	0.42	0.04
	7	63	0.31	0.03

## Discussion

This technical report describes a novel approach to the short-term estimation of census and ventilators needed at hospitals both when facing a surge of patients with COVID-19 and afterward, following the crest of resource requirements. The model slightly underestimates resource requirements during upsurges in admissions rates; this can be dealt with by adding to the estimate one additional patient per day. For example, for the seven-day prediction horizon, one would add seven patients to the predicted census. Similarly, a requirement for an additional 0.5-1 ventilator per day beyond the predicted values can be added during times when the number of patients being placed on mechanical ventilation is increasing. Because of the long lengths of hospital stay, adjustments to the model when the patient census and ventilators in use are steadily dropping probably can be ignored.

A strength of the model is that it uses local hospital data for predictions, and the calculation of the model parameters is straightforward. Such an approach is necessary because distributions for the length of stay or of mechanical ventilation from one hospital cannot accurately be applied to another hospital. Intubation rates among hospitalized patients also will vary among hospitals. We think that such data should be easily obtainable by hospital information systems analysts, as only the COVID-19 status, the duration of hospitalization, and the interval of mechanical ventilation are required.

Applying such one-time changes to the model parameters is simple, as this only involves recalculating the shape and scale of the Weibull fits of the lengths of hospital stay and duration of mechanical ventilation, and adjusting the fraction of patients who required mechanical ventilation. We anticipate that this will take 15 minutes every one to two months. We have been updating the online software as indicated by new knowledge, with maintained version control.

## Conclusions

We present a novel approach for hospitals to model their short-term (i.e., up to one week) requirements related to patient census and the number of ventilators needed both during surge phases of the COVID-19 pandemic and during post-acute phases of the crisis. The provided software can be modified easily by users as desired, with instructions provided on changing the parameter estimations and modeled fits of durations of care. A full assessment of the predictive error of the model will require analysis from multiple other hospitals. However, in the absence of any other current approach at the individual hospital level, we think that the model projections are sufficiently accurate to provide contemporaneous guidance. We hope that our description and posting of the Excel workbook will accelerate the assessment of the utility of this preliminary model in enhancing hospitals’ ability to better manage resource needs during the COVID-19 global health crisis.
